# Noise correlations in the human brain and their impact on pattern classification

**DOI:** 10.1371/journal.pcbi.1005674

**Published:** 2017-08-25

**Authors:** Vikranth R. Bejjanki, Rava Azeredo da Silveira, Jonathan D. Cohen, Nicholas B. Turk-Browne

**Affiliations:** 1 Department of Psychology, Princeton University, Princeton, NJ, United States of America; 2 Princeton Neuroscience Institute, Princeton University, Princeton, NJ, United States of America; 3 Department of Psychology, Hamilton College, Clinton, NY, United States of America; 4 Department of Physics, Ecole Normale Supérieure, Paris, France; 5 Laboratoire de Physique Statistique, École Normale Supérieure, PSL Research University; Université Paris Diderot Sorbonne Paris-Cité; Sorbonne Universités UPMC Univ Paris 06; CNRS, Paris, France; 6 Department of Psychology, Yale University, New Haven, CT, United States of America; Medical Research Council, UNITED KINGDOM

## Abstract

Multivariate decoding methods, such as multivoxel pattern analysis (MVPA), are highly effective at extracting information from brain imaging data. Yet, the precise nature of the information that MVPA draws upon remains controversial. Most current theories emphasize the enhanced sensitivity imparted by aggregating across voxels that have mixed and weak selectivity. However, beyond the selectivity of individual voxels, neural variability is correlated across voxels, and such noise correlations may contribute importantly to accurate decoding. Indeed, a recent computational theory proposed that noise correlations enhance multivariate decoding from heterogeneous neural populations. Here we extend this theory from the scale of neurons to functional magnetic resonance imaging (fMRI) and show that noise correlations between heterogeneous populations of voxels (i.e., voxels selective for different stimulus variables) contribute to the success of MVPA. Specifically, decoding performance is enhanced when voxels with high vs. low noise correlations (measured during rest or in the background of the task) are selected during classifier training. Conversely, voxels that are strongly selective for one class in a GLM or that receive high classification weights in MVPA tend to exhibit high noise correlations with voxels selective for the other class being discriminated against. Furthermore, we use simulations to show that this is a general property of fMRI data and that selectivity and noise correlations can have distinguishable influences on decoding. Taken together, our findings demonstrate that if there is signal in the data, the resulting above-chance classification accuracy is modulated by the magnitude of noise correlations.

## Author summary

A central challenge in cognitive neuroscience is decoding mental representations from patterns of brain activity. With functional magnetic resonance imaging (fMRI), multivariate decoding methods like multivoxel pattern analysis (MVPA) have produced numerous discoveries about the brain. However, what information these methods draw upon remains the subject of debate. Typically, each voxel is thought to contribute information through its selectivity (i.e., how differently it responds to the classes being decoded), with improved sensitivity reflecting the aggregation of selectivity across voxels. We show that this interpretation downplays an important factor: MVPA is also highly attuned to noise correlations between voxels with opposite selectivity. Across several analyses of an fMRI dataset, we demonstrate a positive relationship between the magnitude of noise correlations and multivariate decoding performance. Indeed, voxels more selective for one class, or heavily weighted in MVPA, tend to be more strongly correlated with voxels selective for the opposite class. Furthermore, using a model to simulate different levels of selectivity and noise correlations, we find that the benefit of noise correlations for decoding is a general property of fMRI data. These findings help elucidate the computational underpinnings of multivariate decoding in cognitive neuroscience and provide insight into the nature of neural representations.

## Introduction

The development of fMRI has made it possible to observe the human brain noninvasively as it responds to stimuli or engages in cognitive tasks. For example, participants might be presented with a series of stimuli drawn from two or more categories (e.g., faces and scenes), while the blood oxygenation level-dependent (BOLD) contrast is measured over time from tens of thousands of volumetric pixels (voxels). Different events in the experiment can then be linked to changes in BOLD activity, permitting inferences about the neural basis of cognition (in the example above, about category-selective object perception). However, this is a challenging endeavor because both the physiological processes underlying BOLD activity and the measurement of BOLD activity with fMRI are noisy, and because the resulting datasets can be large and statistically complex [1, 2].

Traditionally, fMRI analyses have focused on the information contained in the timecourse of individual voxels or regions. Such methods are “univariate” because they seek to relate experimental events to single dimensions of BOLD variability, such as the activity averaged across voxels in a region of interest (ROI). Univariate methods have long been the dominant approach when using brain-imaging data to draw inferences about the neural basis of different aspects of cognition [3], including: object perception [4], episodic memory [5], and cognitive control [6, 7]. However, given that cognitive processes are often realized in highly distributed [8] and dynamic [2] ways in the brain, and given that fMRI data have considerable spatial resolution and thus natively live in a high-dimensional space [9], performance achievable with univariate methods may be inherently limited.

A different class of analyses, multivariate pattern analysis (MVPA), was developed to examine such complex neural representations, treating patterns of BOLD activity across voxels and their link to experimental events as a classification problem [1, 10]. MVPA involves training a simple statistical model, in a supervised fashion, to extract regularities in patterns of BOLD activity obtained from different experimental conditions. The trained model is then used to classify or decode the condition under which previously unanalyzed test data were obtained. MVPA has led to a wide range of discoveries about the human brain that often go beyond those achievable by applying univariate methods to the same data, including about: perception [11, 12], attention [13–15], memory [16–19], language processing [20, 21] and decision-making [22, 23].

Although MVPA has been successful across a range of applications, *why* it is successful has been harder to pin down [10, 24, 25]. One early and still prominent proposal is that MVPA is sensitive to local biases in the manner in which sub-voxel information is represented across populations of voxels [8, 11, 12]. For example, orientation information in the primary visual cortex is represented in sub-millimeter columns [26, 27] and thus would be obscured at the level of voxels, which typically span a couple of millimeters. However, because the distribution of orientation columns across voxels is irregular, any given voxel may have a random over-representation of, and thus a weak bias toward, a particular orientation. Prior studies have argued that by aggregating such weak biases across a population of voxels, the orientation of a stimulus can be reliably decoded using MVPA [11]. Another possibility is that MVPA allows for the identification of information represented at a larger scale that spans multiple, spatially disparate voxels. For instance, it is possible to decode stimulus orientation based on the systematic way in which areas of retinotopic visual cortex over-represent the orientation perpendicular to the radius from the fovea [28].

Regardless of the scale of neural representations, the assumption underlying this prior work is that considering patterns of activity across voxels rather than averaging over them (as in univariate ROI analyses, for example) provides additional or different sensitivity. These theories view neural representations as points in a high-dimensional activity space, with each voxel in the pattern representing a potentially informative dimension. Although two stimulus categories may be hard to distinguish along any one dimension in this space, jointly considering many voxels allows for better inference by exploiting more dimensions of information.

This interpretation of MVPA downplays an important factor known to influence the representation of information in populations of neurons—that neural variability is correlated *in vivo* [29–33]. Both experimental [29, 30, 32] and computational [34–36] studies have shown that correlations in neural variability have a significant impact on the information content of neural populations; see [37, 38] for reviews. More relevant for present purposes, accurate decoding depends on taking such noise correlations into account [39, 40].

Given that noise correlations are important for neural decoding, they may also influence decoding of fMRI data. Indeed, noise correlations amongst voxels are widespread in fMRI, both during rest [41, 42] and in the background of tasks [43, 44], driven in part by anatomical connections [45]. Yet, prevailing interpretations of why multivariate decoding is effective have not sufficiently acknowledged the relevance of noise correlations to the decoding of information from populations of voxels. This is not to say that the classification algorithms themselves disregard correlations among voxels. Indeed, in most cases these algorithms are sensitive to the presence of correlations [46], and decoding performance is influenced by them. Our argument is instead that prevailing interpretations of why MVPA is effective generally center on the benefits of aggregating the information conveyed by patterns of mean activity across voxels, and overlook the influence of correlations. Even when theories have explicitly considered the influence of correlations, they have generally considered *signal* correlations: moment-to-moment correlations in the representation of task-dependent stimulus information across multiple voxels in the population (i.e., overlap in the representation of the underlying signal across multiple voxels). For instance, if two voxels contain the same signal across training patterns, classification algorithms such as support vector machines (SVM) and regularized logistic regression can assign one voxel a higher weight than the other [47].

Here we propose that *noise* correlations—which exist persistently before, during, and after experimental events—help explain the effectiveness of MVPA. In contrast to signal correlations, noise correlations reflect the extent to which noise in the activity of a voxel is correlated with noise in the activity of other voxels in the population. The theory that motivates this hypothesis is from a recent computational study [36]. This study showed that the impact of noise correlations on multivariate decoding depends on whether the correlations are between neurons from homogeneous vs. heterogeneous populations, with the latter being beneficial and the former being detrimental. When considering homogeneous populations—neurons that code for the same stimulus variable—decoding performance worsens as noise correlations increase. That is, when neurons in a population are selective for the same stimulus, lower noise correlations between them allow the decoder to exploit more dimensions of information. Indeed, experimental [29, 30, 32] and computational studies [48] have found a relation between lower noise correlations in homogeneous populations and increased information.

Importantly, in contrast to homogeneous populations, decoding performance for heterogeneous populations of neurons that code for different stimulus variables can improve as noise correlations increase [36]. The intuition is that, given a constant amplitude of noise, the presence of noise correlations between neurons coding for different stimulus variables allows a multivariate decoder to recognize that the correlated (or shared) variance can be attributed to dimensions that are irrelevant for discriminating between the variables, and can thus be ignored. This reduces the dimensionality of the classification problem and, more importantly, the amount of overlap between the categorical distributions, thereby improving performance [49]. Indeed, a recent theoretical study [46] similarly argued that weight vectors in decoding models, such as MVPA, are influenced by both the signal and noise in brain imaging data, thereby suggesting a similar influence of heterogeneous noise correlations on classification performance.

**Fig 1:**
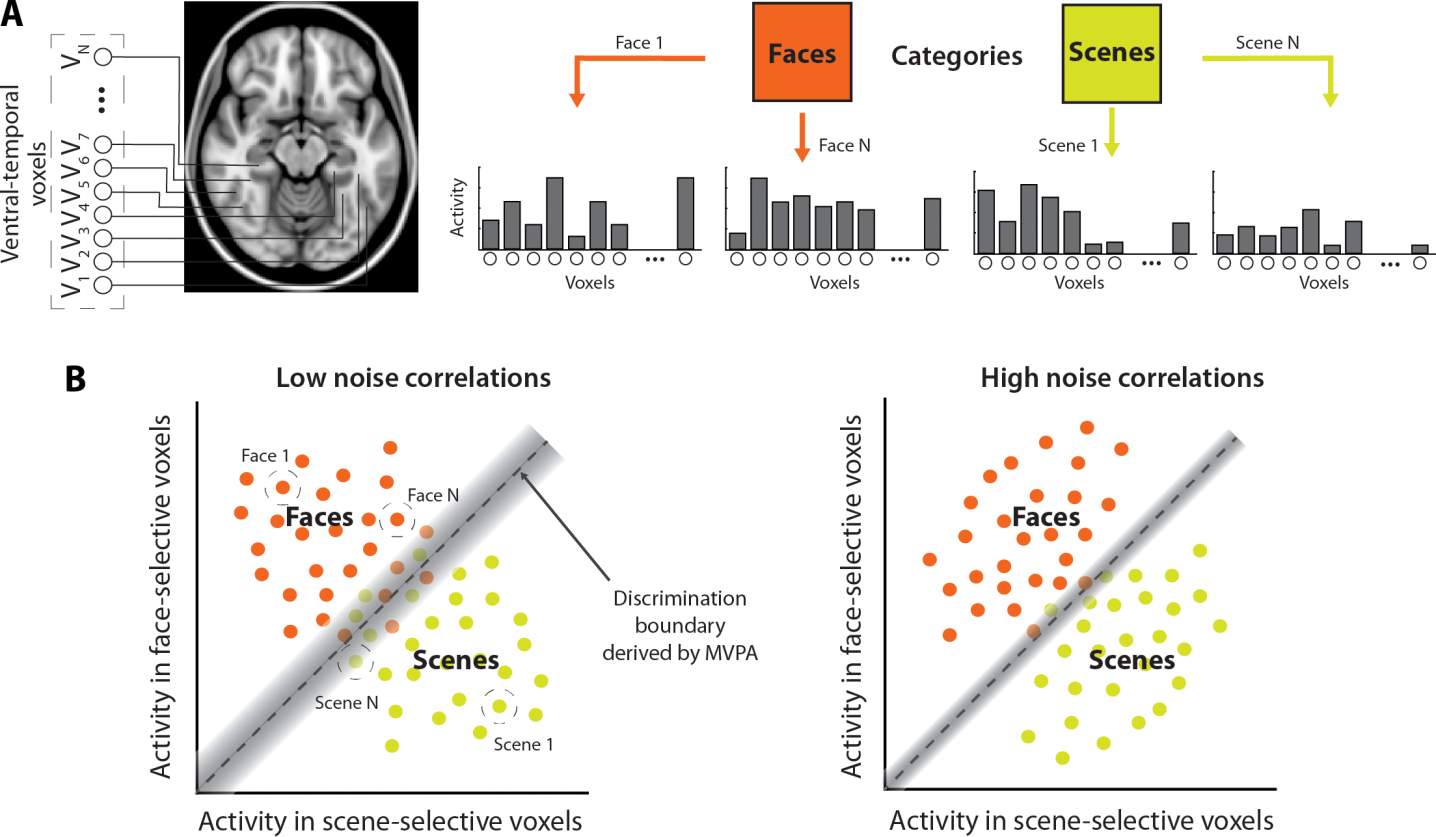
An illustration of the influence of noise correlations on face/scene MVPA decoding. (A) In a typical experiment, participants may be presented with a series of stimuli drawn from two or more categories (e.g. faces and scenes), while fMRI BOLD activity (illustrated here by gray bars) is measured in ventral temporal voxels, some of which exhibit a preference for faces (face-selective voxels), and others for scenes (scene-selective voxels). (B) Multivariate decoding methods such as MVPA seek to find a decision boundary (gray dashed line) in the high-dimensional space of voxel activity patterns (collapsed here, for illustrative purposes, to a 2-D space with activity across face-selective voxels on the ordinate, and activity across scene-selective voxels on the abscissa). Due to variability in BOLD activity, each category is represented by a distribution in this space, and classification errors result from overlap in these distributions (shaded region). When voxels selective for one of the categories have high noise correlations with voxels selective for the other category (scenario illustrated on the right), activity distributions can be elongated in the direction parallel to the discrimination boundary, resulting in reduced overlap (smaller shaded region) and improved classification accuracy.

Here we extend this theory—developed [36] and supported [50–52] at the level of neurons—to populations of voxels in fMRI (Fig 1). Two challenges arise from this extension: First, it is impossible to know whether a given voxel contains a homogenous neuronal population and even whether multiple voxels with similar selectivity can be considered truly homogenous. Thus, we focus on the theoretical predictions associated with decoding from heterogeneous populations (i.e., that noise correlations among voxels selective for different stimuli will improve decoding of these stimuli). Second, the theory was developed to account for the influence of positive noise correlations. However, negative correlations can arise in fMRI (e.g., depending on preprocessing steps), so our analyses consider the influence of both positive and negative noise correlations.

We find that MVPA decoding performance is influenced not only by the selectivity of individual voxels but also by noise correlations between heterogeneous populations of voxels. Across several analyses of an fMRI dataset, we demonstrate a positive relationship between the magnitude of noise correlations and decoding performance, and we show that as expected with such classifier algorithms [46, 49], MVPA exploits noise correlations by assigning higher weights to voxels with higher noise correlations. We also show that selectivity and noise correlations influence decoding in a complementary fashion—as long as there is signal in the data, performance is modulated by the magnitude of noise correlations. Indeed, voxels that were highly selective for one class also exhibited higher noise correlations with voxels selective for the other class. Finally, using a simple model of BOLD activity, we simulate different levels of selectivity and noise correlations in artificial data and show that the benefit of noise correlations for decoding is a ubiquitous property of fMRI data beyond the example dataset.

## Results

### Overview

We used a subset of the data from an fMRI study on attentional control [53]. Seventeen participants were presented with blocks of face or scene stimuli interleaved with blank periods during two “localizer” runs. In addition, data were collected during two “rest” runs in which participants only fixated a central point. Using one of the localizer runs, we fit a general linear model (GLM) to the activity observed in ventral temporal cortex, and labeled each voxel as either face-selective or scene-selective based on whether that voxel had greater activation in response to the presentation of face vs. scene stimuli. Then, we used the rest runs to compute noise correlations, since there were no stimuli or tasks in these runs. We were specifically interested in heterogeneous noise correlations (i.e., noise correlations between voxels with different selectivity) and thus calculated, for every voxel, the average correlation between its timecourse and the timecourse of all voxels selective for the opposite category. Finally, to examine how these noise correlations influenced decoding performance, we selected voxels from both face- and scene-selective populations with either high or low noise correlations, and used the other, separate localizer run to train and cross-validate a multi-way (face/scene/blank) classifier based on the patterns of activity from these voxels.

### Decoding performance

**Fig 2:**
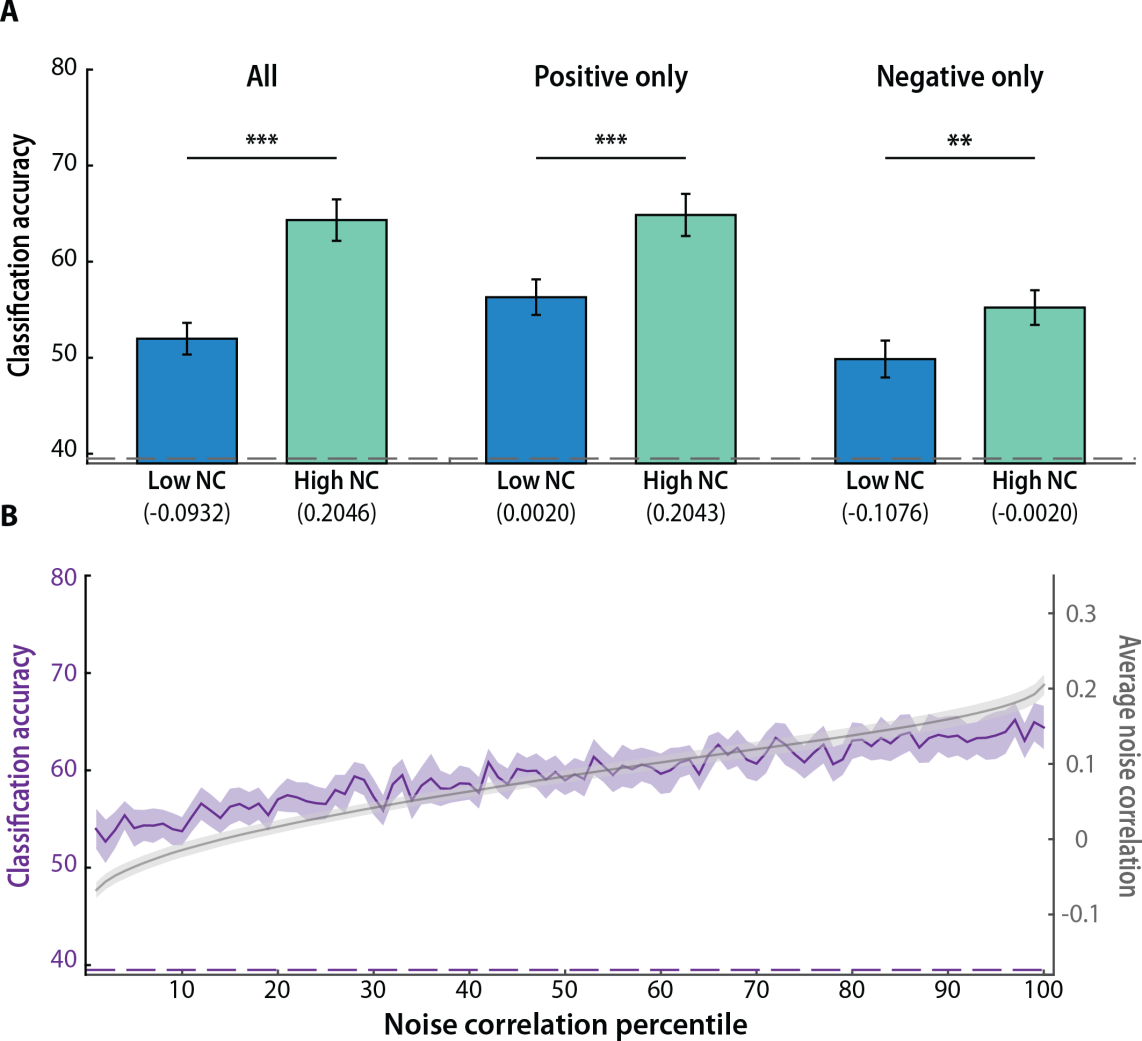
Noise correlations and MVPA decoding. (A) Classification accuracy was better for patterns of activity over voxels with high (top 1%) vs. low (bottom 1%) noise correlations in the raw distribution, and the positive values from the raw distribution; the same pattern held for negative values from the raw distribution, but with high and low defined as the top and bottom 6%, respectively (to accommodate the smaller sample of negative correlations). Columns represent means and error bars represent SEM across participants. The number below each column is the average noise correlation, across the voxels in the selected set and across all participants, provided for descriptive purposes. The dashed gray line denotes the baseline “chance” level of classification accuracy obtained by permuting the class labels 10,000 times. The classifier was trained on three classes (face, scene, and blank), but chance is not 33% because there were more blank samples. (B) Classification accuracy improved monotonically with an increase in the magnitude of noise correlations. The solid purple line represents mean classification accuracy in every percentile of voxels, and the ribbon represents SEM across participants. The solid gray line represents mean noise correlations in every percentile (for descriptive purposes, as this was the basis of sorting), and the ribbon represents SEM across participants. The dashed purple line denotes the empirically defined chance level of classification accuracy obtained from the permutation analysis. ***p < 0.001, **p < 0.01.

If MVPA is sensitive to noise correlations, then classification accuracy should be better for patterns of activity from voxels that are strongly vs. weakly correlated with voxels selective for the opposite category. As a first pass, we focused on voxels with the highest vs. lowest 1% of noise correlations (Fig 2A) and found that classification was better for voxels with the highest noise correlations (*t*_*16*_ = 7.24, *p* < 0.0001). This sorting was based on raw values (high more positive, low more negative), but the same result was obtained when we analyzed positive correlations (high more positive, low closer to zero; *t*_*16*_ = 4.12, *p* < 0.001) and negative correlations (high closer to zero, low more negative [54]; *t*_*16*_ = 3.19, *p* < 0.01).

For a more continuous sense of this relationship, we divided voxels into percentiles of raw noise correlations (Fig 2B). Classification accuracy improved monotonically as MVPA was applied to voxel sets with greater noise correlations (slope vs. 0: *t*_*16*_ = 6.66, *p* < 0.0001). Taken together, these results demonstrate a clear influence of the magnitude of heterogeneous noise correlations on decoding performance.

### Influence of bin size

**Fig 3:**
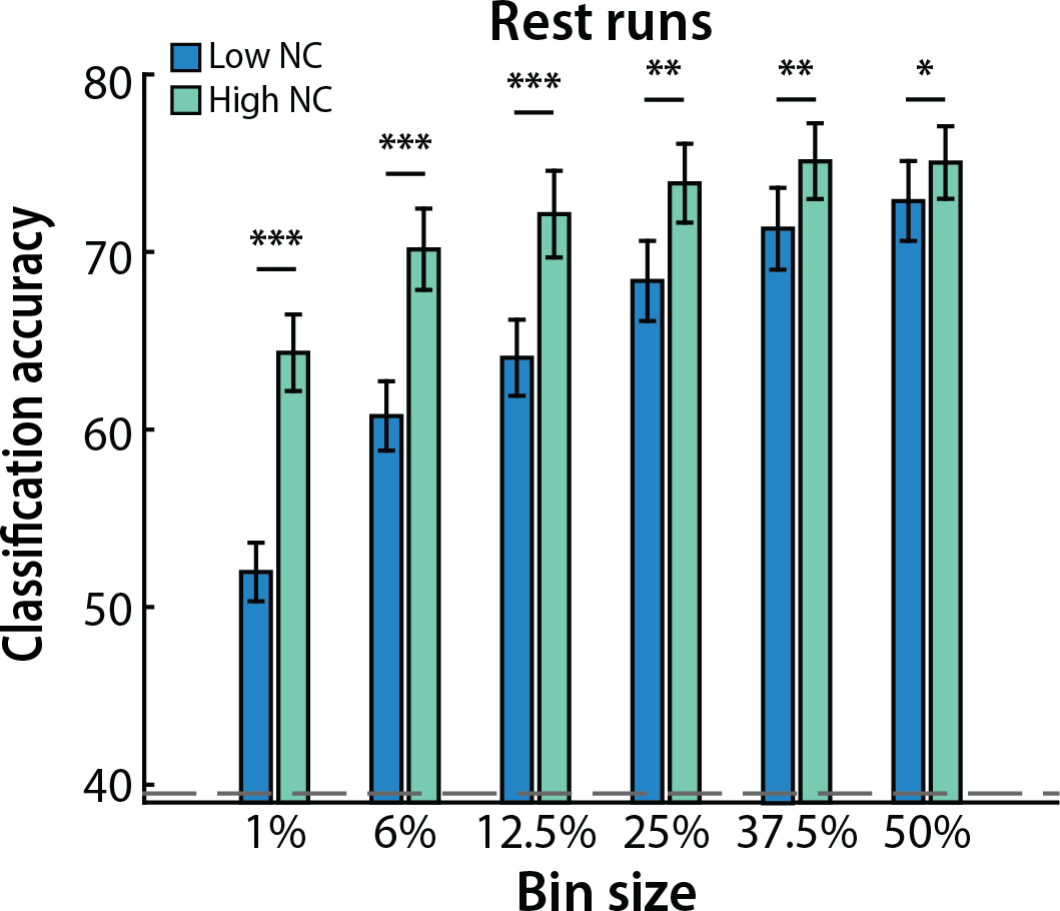
Number of voxels. Classification accuracy was consistently better for patterns of activity over an increasing number of voxels with high (green) vs. low (blue) noise correlations, with a larger difference for smaller bin sizes. Columns represent means and error bars represent SEM across participants. The dashed gray line denotes permuted chance. ***p < 0.001, **p < 0.01, *p < 0.05.

We chose an arbitrary, small bin size of voxels (1%) in the analyses above. To examine how this parameter affected our findings, we repeated the analysis of raw values with larger bin sizes of high and low noise correlations: 6%, 12.5%, 25%, 37.5% and 50% (Fig 3). While overall decoding performance improved with increasing bin size, decoding was consistently better for patterns of activity from voxels with high vs. low noise correlations (*p*s < 0.02). A 2 (noise correlation magnitude: high vs. low) x 6 (bin sizes) repeated-measures ANOVA revealed that the difference was greater for smaller bin sizes: In addition to main effects of noise correlation magnitude (F_1,16_ = 28.57, *p* < 0.0001) and bin size (F_5,80_ = 164.30, *p* < 0.0001), there was a reliable interaction between these variables (F_5,80_ = 14.12, *p* < 0.0001). This interaction is also consistent with the monotonic relationship across percentiles reported above (Fig 2B): As bin size increased, both the high and low sets included more voxels with intermediate magnitudes of noise correlation, thereby bringing performance closer to the mean across magnitudes.

### Influence of regularization

The analyses above use an L2-norm regularized logistic regression classifier for MVPA. Such regularization helps avoid over-fitting—which was a risk given that the number of samples in the training set was much smaller than the number of voxels whose weights were learned—by constraining the learning process. In the case of L2-norm regularization, the sum of squares of the voxel weights is penalized (here, penalty parameter = 1). Because all voxels contribute to this sum, this regularization induces interactions between voxels when determining weights. It could be possible that the influence of noise correlations on decoding performance reflects their effects on such interactions *per se* rather than the placement of the classifier boundary. To evaluate this possibility, we repeated the bin size analysis with regularization turned off. Classification accuracy decreased across the board (presumably because of over-fitting), but we still found greater accuracy for high vs. low noise correlations (S1 Fig). This suggests that the benefit of noise correlations was not an artifact of regularization.

### Task-dependence of noise correlations

So far, we have calculated noise correlations from the rest runs and performed classification on the localizer runs. In using a different run to compute noise correlations, we tacitly assumed that they were stationary across rest and localizer runs. However, noise correlations may depend on the task condition or may be most closely tied to decoding when actually obtained from the data being decoded. To examine this possibility, we computed noise correlations between voxels during the localizer run used for crossvalidation. This is challenging because stimulus-evoked responses can induce signal correlations. Thus, we first regressed out these responses (and global noise sources) and examined BOLD correlations in the residuals. This “background connectivity” approach has been used successfully across a range of tasks to study noise correlations [44, 55–57].

**Fig 4:**
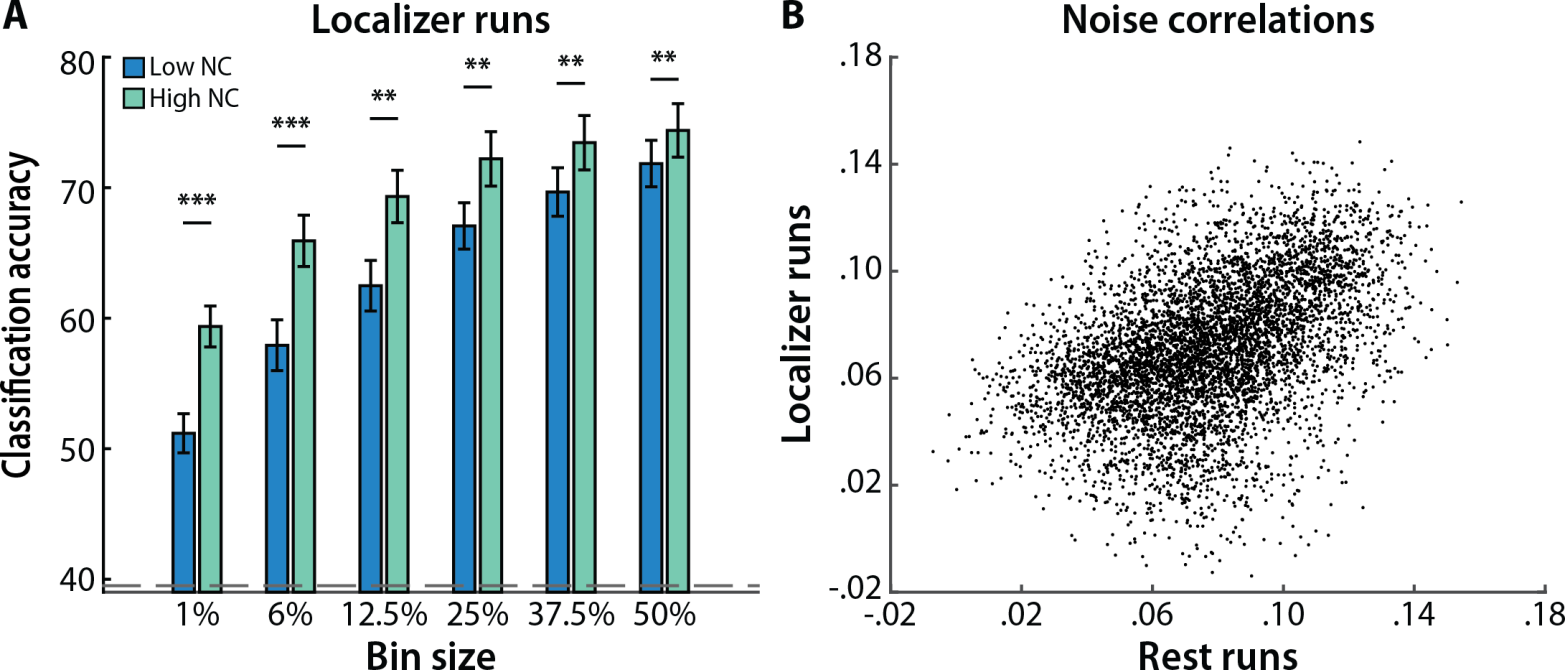
Background noise correlations. (A) Noise correlations calculated from localizer runs had a similar effect on MVPA as noise correlations computed from rest runs. Classification accuracy was again better for patterns of activity over voxels with high (green) vs. low (blue) noise correlations, with a similar interaction by bin size. Columns represent means and error bars represent SEM across participants. The dashed gray line denotes permuted chance. (B) The noise correlations calculated from rest runs were similar to the noise correlations calculated from localizer runs. Each dot represents one voxel, with its two coordinates reflecting the heterogeneous noise correlation (i.e., average correlation with voxels with opposite selectivity) from rest and localizer runs, respectively, averaged across participants for purposes of visualization. ***p < 0.001, **p < 0.01.

We again identified face- and scene-selective voxels from one localizer run, but then calculated heterogeneous noise correlations (i.e., in the residuals) and classified the other localizer run. The pattern of results was nearly identical to that obtained when noise correlations were calculated from the separate rest runs, as seen by repeating the bin size analysis (Fig 4A). Classification accuracy was again consistently better for high vs. low noise correlations (*p*s < 0.01), and there were main effects of noise correlation magnitude (F_1,16_ = 18.28, *p* < 0.001) and bin size (F_5,80_ = 152.77, *p* < 0.0001), and an interaction (F_5,80_ = 6.78, *p* < 0.0001). In fact, the heterogeneous noise correlation for a given voxel was fairly stable across rest and localizer runs (Fig 4B). This was quantified with Spearman’s rank order correlation across voxels within participant (mean rho = 0.21; *t*_16_ = 5.30, *p* < 0.0001). Given these results, and because the rest dataset was fully separate, we returned to using the rest runs for calculating noise correlations in the remaining analyses.

### Comparison to random

We next compared the classification accuracy obtained by selecting voxels with high or low noise correlations in the rest runs across the six bin sizes to classification accuracy obtained for sets of voxels of equal size chosen randomly (irrespective of noise correlation). If MVPA automatically exploits noise correlations in a given population of voxels, as long there are enough voxels in the population with high noise correlations, MVPA should assign high weights to these voxels and achieve similar performance to a classifier trained only on voxels with high correlations.

**Fig 5:**
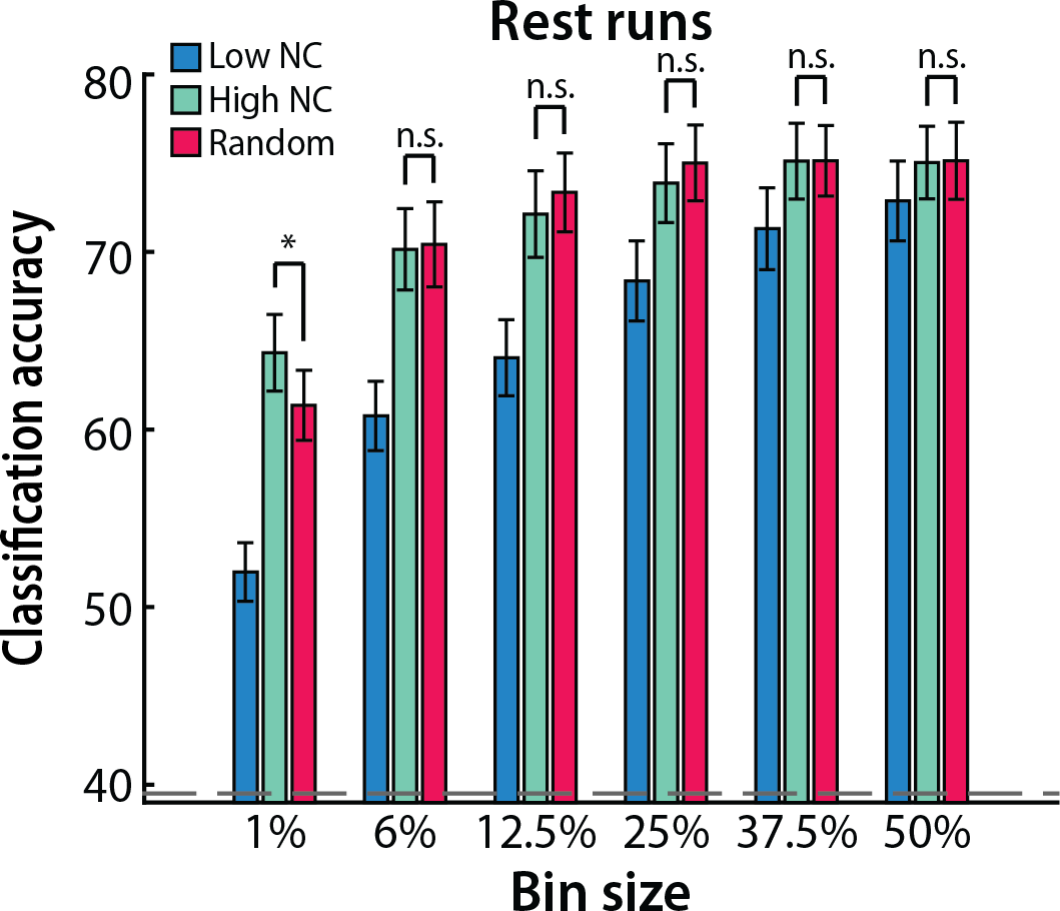
Randomly selected voxels. Classification accuracy was similar for voxels selected for having high noise correlations (green) vs. voxels selected randomly (red), except for the 1% bin size where the high noise set outperformed the random set. Across all bin sizes, classification accuracy was the lowest for voxels selected for having low noise correlations (blue). Columns represent means and error bars represent SEM across participants. *p < 0.05.

For the smallest bin size of 1%, the high noise correlation set produced better decoding performance than the random set (*t*_*16*_ = 2.38, *p* = 0.03), consistent with the notion that there were not enough voxels with high noise correlations in the random set (Fig 5). However, starting at the 6% bin size, decoding performance was indistinguishable between high noise correlation and random sets (*p*s > 0.09). Critically, highlighting the efficiency of MVPA at exploiting noise correlations, the random sets exceeded the low noise correlation sets at all bin sizes (*p*s < 0.001). Taken together, these results suggest that a small number of voxels with high correlations dominate MVPA decoding performance even when considering large sets of voxels.

### Relationship between classifier weights and noise correlations

We assumed in the previous analysis that MVPA as typically applied (i.e., without explicitly considering noise correlations during feature selection) performed as well as MVPA over voxels with high noise correlation because it automatically assigned these voxels higher weights. Here we test this directly by carrying out MVPA over all ventral temporal voxels without feature selection and examining the relationship between assigned classifier weights and average heterogeneous noise correlations. That is, if a voxel was determined to be face-selective in one localizer run, how correlated was (a) its average noise correlation with scene voxels in the rest runs, with (b) its weight assigned for the face category in a classifier trained on the second localizer run?

**Fig 6:**
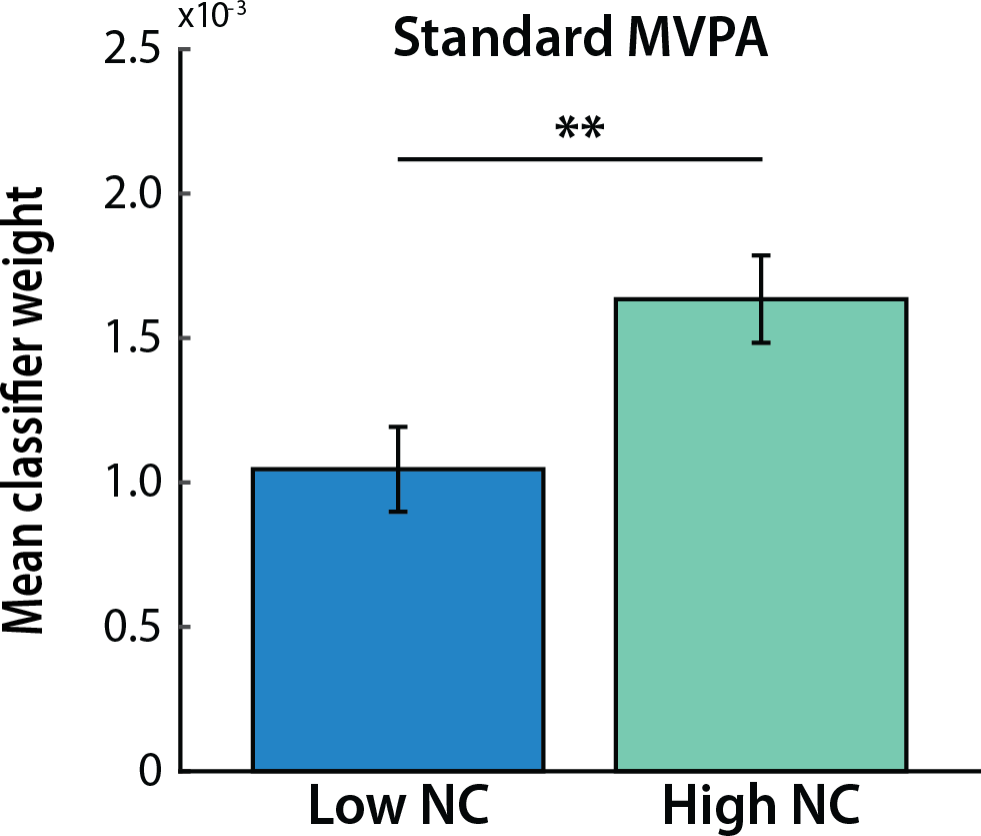
Classifier weights. A median-split analysis revealed that voxels with higher noise correlations were assigned higher weights than voxels with lower noise correlations. Columns represent means and error bars represent SEM across participants. **p < 0.01.

We first summarize this relationship using a median-split analysis on the noise correlations (Fig 6), which revealed that voxels with higher noise correlations were assigned higher weights (*t*_*16*_ = 3.96, *p* = 0.001). Another way to look at this relationship is to calculate the Spearman rank order correlation between noise correlation and classifier weight across voxels. This correlation was reliable across participants (mean rho = 0.045; *t*_*16*_ = 3.58, *p* = 0.002).

### Influence of selectivity

The analyses above demonstrate that MVPA decoding performance is enhanced when voxels with high vs. low noise correlations (measured during rest or in the background of the task) are selected during classifier training, and that voxels which receive high classification weights in MVPA tend to exhibit high noise correlations with voxels selective for the other class being discriminated against. However, in addition to the magnitude of noise correlations, decoding performance is also influenced by the selectivity of individual voxels (i.e., how differently a voxel responds to the conditions being classified). In this section, we examine the relative influence of selectivity on MVPA decoding performance.

**Fig 7:**
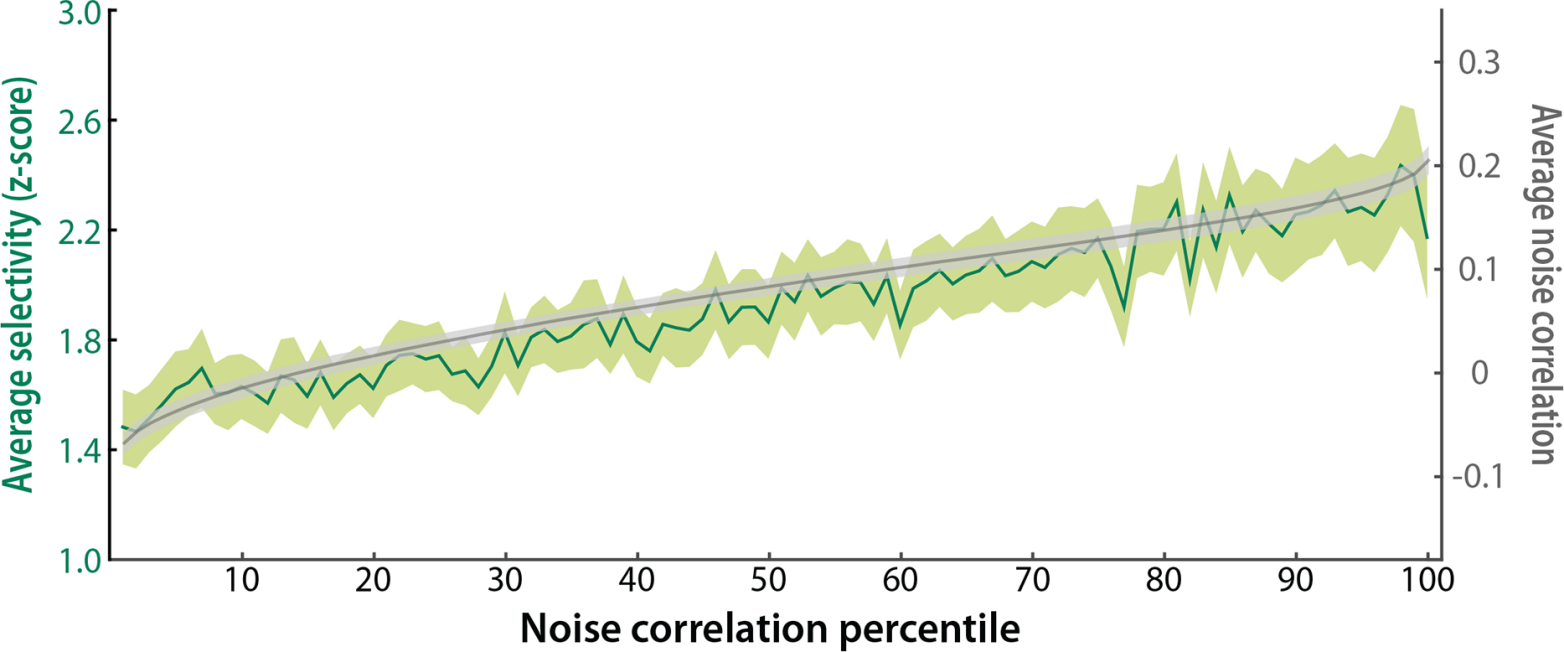
Selectivity and noise correlations. Average selectivity increased monotonically as the magnitude of noise correlations increased. The solid green line represents mean selectivity in every percentile of voxels, and the ribbon represents SEM across participants. The solid gray line represents mean noise correlations in every percentile (for descriptive purposes, as this was the basis of sorting), and the ribbon represents SEM across participants. Each percentile included the same voxels as in Fig 2B.

We first consider the extent to which selectivity and noise correlations interact. For instance, when we divided voxels in our dataset into percentiles of raw noise correlations, we observed a monotonic improvement in MVPA decoding performance with an increase in the magnitude of noise correlations (Fig 2B). How does selectivity vary across these sets of voxels? To answer this question, we took the absolute value of the selectivity scores that had been used to identify face and scene voxels in one localizer run (i.e., for determining which voxels should count as having opposite selectivity when calculating heterogeneous noise correlations). As a reminder, these scores reflect the face vs. scene contrast from the GLM, specifically the z-scored difference of the parameter estimates modeling the average evoked response from face and scene blocks, respectively. Average selectivity increased monotonically (Fig 7) as we moved from voxels with low noise correlations to voxels with high noise correlations (slope vs. 0: *t*_*16*_ = 5.03, *p* < 0.001), and the Spearman rank order correlation between noise correlation and selectivity across voxels was reliable (mean rho = 0.076; *t*_*16*_ = 4.17, *p* < 0.001). In other words, voxels with higher selectivity for one of the two categories also had higher noise correlations with voxels selective for the other category.

**Fig 8:**
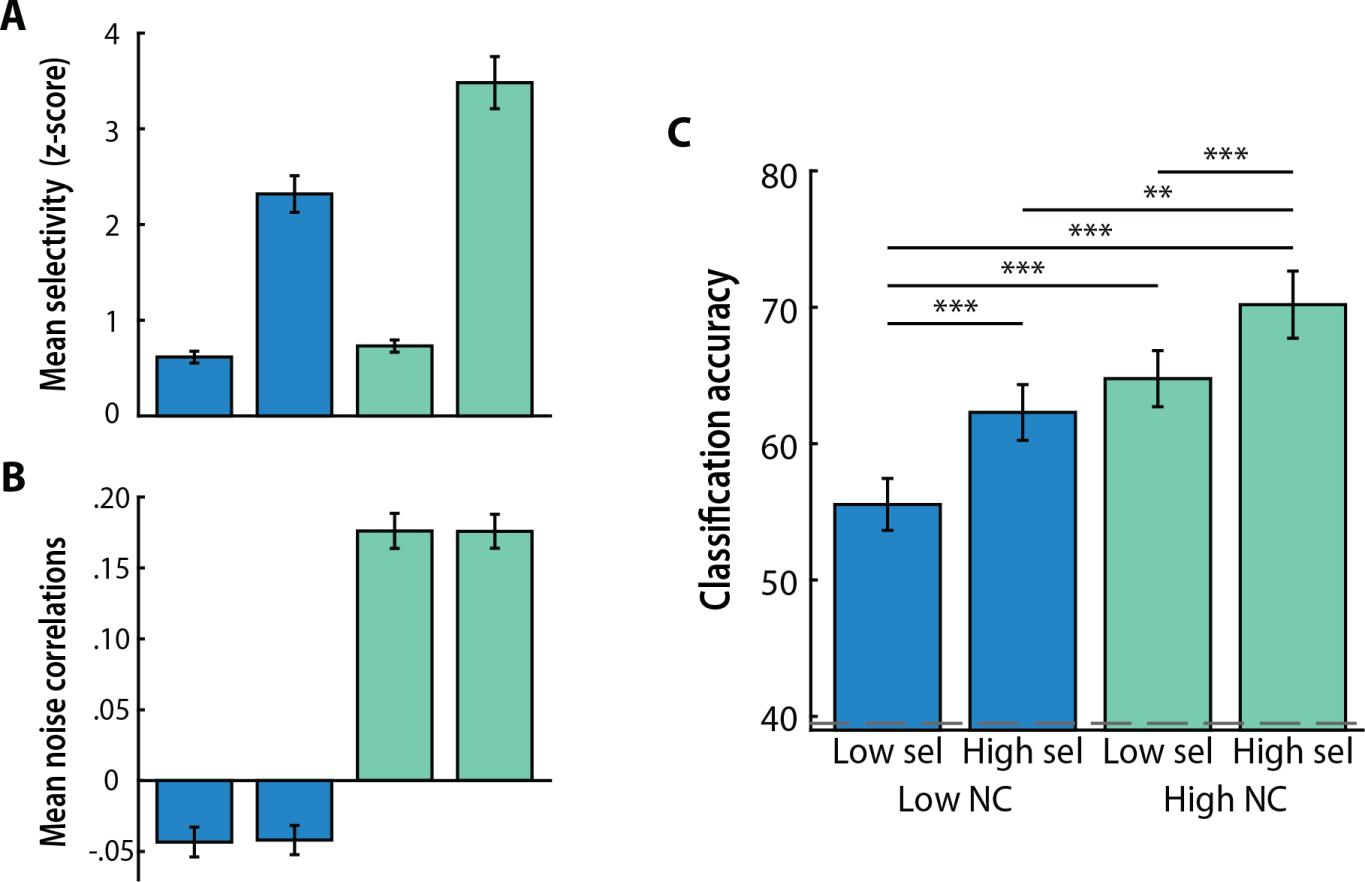
Cumulative influence of selectivity and noise correlations. Classification accuracy for voxels selected for having either low noise correlations and low or high selectivity (blue) or high noise correlations and low or high selectivity (green). Mean selectivity (A) and noise correlations (B) across voxels in each set. (C) Noise correlations can influence classification performance in a complementary manner to selectivity. The dashed gray line denotes chance. Columns represent means and error bars represent SEM across participants. Significance of pairwise comparisons is depicted here. ***p < 0.001, **p < 0.01.

Given the link between selectivity and noise correlations across voxels in our empirical dataset, we next sought to examine their cumulative influence on decoding. We selected voxels with the top vs. bottom 12% of noise correlations, and within each set selected voxels with high vs. low selectivity based on a median split of voxel selectivity from the GLM. We then examined MVPA classification accuracy for the patterns of activity from voxels in each of the resulting four bins with 6% of voxels (Fig 8).

Of particular note in this analysis is the comparison between low noise correlation/low selectivity and high noise correlation/low selectivity, which had comparable levels of selectivity (1^st^ and 3^rd^ columns of Fig 8A) but dramatically different classification accuracy (same columns of Fig 8C). This suggests that as long as there is a minimum amount of signal conveyed by selectivity, which allows for above-chance classification, noise correlations can be sufficient to increase decoding performance (same columns of Fig 8B). This claim is further reinforced by the comparison of low noise correlation/high selectivity to high noise correlation/low selectivity. Although there was a dramatic difference in signal conveyed via selectivity (2^nd^ and 3^rd^ columns of Fig 8A), classification accuracy did not differ and was in fact numerically in the opposite direction (same columns of Fig 8C), suggesting that the selectivity difference was offset by the reverse difference in noise correlations (same columns of Fig 8B). Taken together, these results support the notion that when selectivity differences are present, noise correlations can influence classification accuracy.

### Model simulations

So far, we have used an existing fMRI dataset to demonstrate that MVPA is highly attuned to noise correlations between voxels, and that decoding performance may be sensitive to the information carried both by the selectivity of individual voxels and the noise correlations between them. We next sought to expand upon these findings in two ways: First, as described above, selectivity and noise correlations were inherently confounded in the empirical dataset. How might we better examine the cumulative contributions of noise correlations and selectivity to decoding performance? Second, all of the findings reported above were based on one fMRI dataset with particular characteristics. To what extent do our conclusions apply to other datasets and reflect a general principle about the computational underpinnings of MVPA? To address these issues, we developed a simple model of selective coding in the presence of noise correlations, wherein we could independently vary voxel selectivity and heterogeneous noise correlations. By performing MVPA over artificial BOLD activity generated from this model, we could then simulate the influence of different parameters.

The model included a set of voxels roughly matched in number to the 1% bin size in our earlier analyses. By construction, half of the voxels responded preferentially to face stimuli and the other half to scene stimuli. The mean responses, variances, and correlations of all voxels in the model were drawn from the range observed in our empirical dataset, ensuring that the simulated voxels produced physiologically realistic activity. Following Azeredo da Silveira & Berry (2014), we used a Gaussian approximation in each of 100 model “participants” to sample data for time points from face and scene blocks (matched to the number of face and scene TRs in the empirical dataset). To ensure that the resulting timecourses were temporally autocorrelated like real BOLD activity [58], we convolved them with a canonical hemodynamic response function (HRF). Finally, we performed cross-validated MVPA over the artificial patterns of activity obtained from the simulated voxels.

**Fig 9:**
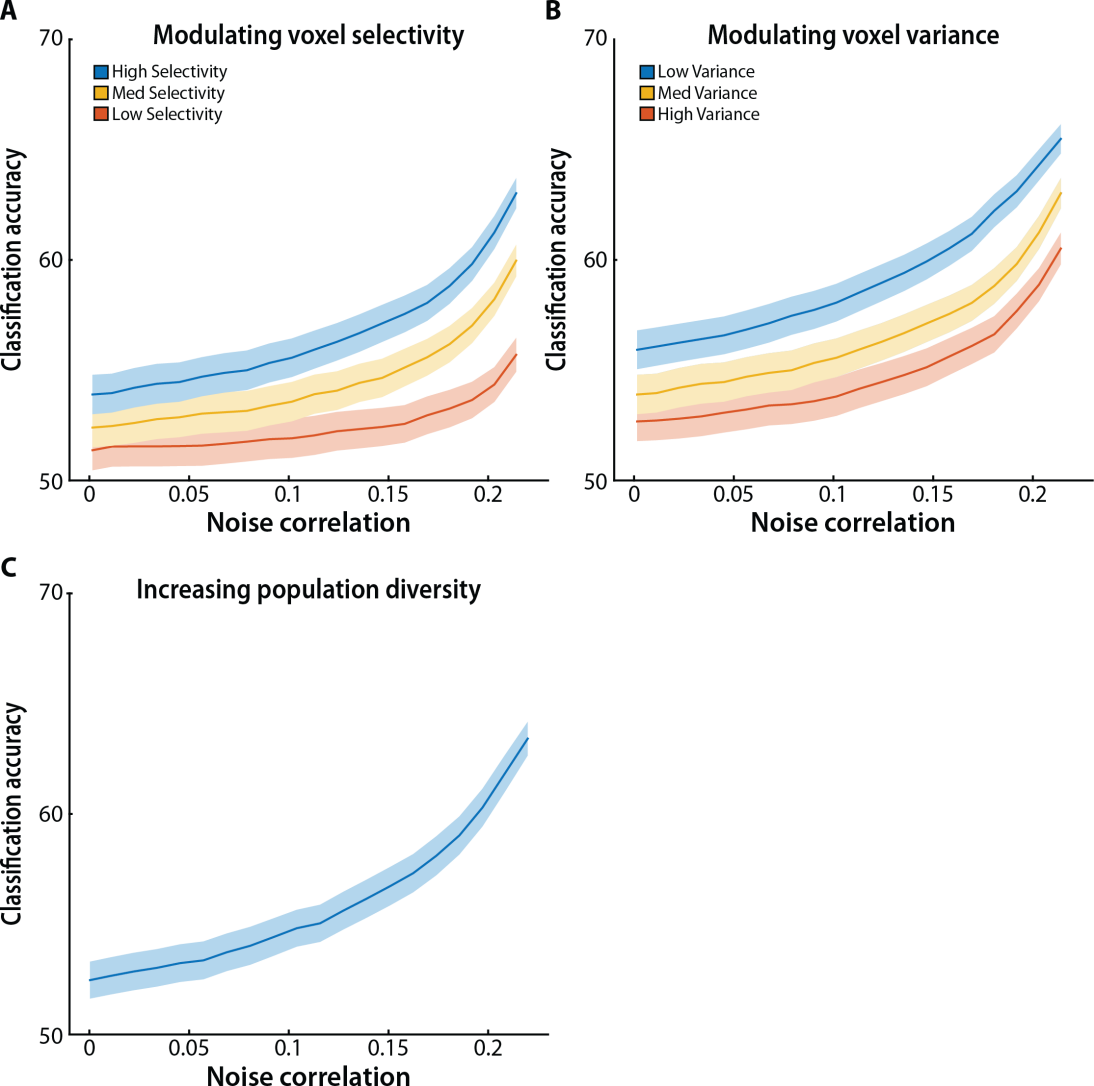
Model simulations. Classification accuracy improved monotonically with an increase in the magnitude of heterogeneous noise correlations in simulated populations of face- and scene-selective voxels. Solid lines represent mean classification accuracy as the magnitude of noise correlations increased, with all other parameters fixed. Ribbons represent SEM across model participants. (A) Overall classification accuracy dropped as voxel selectivity decreased. However, across all selectivity profiles, classification accuracy improved monotonically with an increase in the magnitude of noise correlations. (B) Overall classification accuracy dropped as voxel variance increased. However, across all levels of variance, classification accuracy improved monotonically with an increase in the magnitude of noise correlations. (C) Increasing diversity in the response properties of individual voxels within the simulated face- and scene-selective populations did not qualitatively change the pattern of results. Indeed, increasing population diversity led to a steeper improvement in classification accuracy as a function of noise correlations. See Methods for the parameter values used in each simulation.

We first sought to examine the influence of heterogeneous noise correlations on decoding performance. Noise correlations across pairs of voxels varied according to whether the voxels were drawn from the pool of face-selective voxels, the pool of scene-selective voxels, or one from each of the pools. We performed 20 simulations manipulating the magnitude of across-pool noise correlations linearly between 0 and 0.22 (i.e., the range of positive noise correlations in the empirical dataset), while holding all other parameters constant. There was a monotonic increase in classification accuracy as the magnitude of heterogeneous noise correlations increased (blue curve in Fig 9A). This is precisely the pattern predicted by the computational theory on which our study was based [36], and is similar to the pattern of results observed in our empirical dataset. Notably, by allowing noise correlations to vary while the selectivity of the voxels in the two pools was held constant, these results show that noise correlations are sufficient to influence above-chance decoding performance.

To examine the influence of voxel selectivity, we repeated the analysis above but further manipulated the strength of face and scene selectivity in the mean responses of voxels from the two pools, over a fixed range of noise correlations. As expected, when voxel selectivity decreased across three levels, overall decoding performance also decreased (Fig 9A). However, at all levels, we observed the same monotonically increasing relationship between classification accuracy and the magnitude of noise correlations. Notably, selectivity affected classification accuracy even with near-zero noise correlations, but the effect of selectivity was stronger in the regime of stronger noise correlations.

We next sought to examine the extent to which these results depend on the specific parameters used in our simulations. For instance, in the simulations described thus far, the variance of all voxels was matched to the median variance observed in our empirical dataset. Given that overall noise in the system, correlated or otherwise, is ultimately a function of the variability in the activity of individual voxels, we examined the extent to which our results depended on the magnitude of voxelwise variance. Repeating our analysis across three levels of variance, spanning the range observed in our empirical dataset, we found a similar influence of noise correlations on classification accuracy (Fig 9B). Specifically, as voxel variance increased, thereby increasing noise in the system, overall decoding performance went down; however, at every level of variance, we observed the same relationship between classification accuracy and the magnitude of noise correlations.

Another modeling choice we made was to sample activity within the face- and scene-selective voxel pools based on homogeneous mean, variance, and correlation values matched to population averages from our empirical dataset. We next examined the influence of introducing heterogeneity in the response properties of the simulated population of voxels. We generalized our model to include greater voxelwise diversity by randomly varying the population covariance matrix according to a Gaussian distribution with SD equal to 10% of the original value. We similarly varied the mean responses of individual voxels (while maintaining selectivity) in each population according to a Gaussian distribution with SD matched to the mean within-population SD from our empirical dataset. We obtained the same pattern of results from MVPA, with classification accuracy increasing monotonically as the magnitude of noise correlations increased (Fig 9C). Indeed, greater population diversity led to a steeper increase in classification accuracy, consistent with the notion that heterogeneity can be beneficial, especially at higher levels of noise correlation.

## Discussion

MVPA has proven useful for decoding information from brain imaging data [1, 10], with insights often extending what has been learned from univariate methods. Although the effectiveness of MVPA has been widely acknowledged, which aspects of neural representation MVPA taps into are still debated [2, 10, 24, 25, 28]. Prior theories argued that MVPA benefits from aggregating signals across voxels—either local biases in the mapping of micro-scale representations onto voxels [11, 25] or more global, macro-scale representations that span multiple voxels [28]. In both cases, the argument was that MVPA exploits the distribution of weak or uncertain feature-selective signals to identify regularities that discriminate experimental conditions.

Our findings show that this interpretation is incomplete: Instead of thinking of each voxel as making a distinct contribution to the information represented collectively by the population of voxels, MVPA is also highly attuned to noise correlations between voxels. This reflects the mechanics of classification algorithms [49] and builds on neurophysiological studies showing both that noise correlations impact the information content of neural populations [36–38] and that accurate decoding of this information requires taking these noise correlations into account [39, 40]. Specifically, our study was inspired by a recent computational theory [36], which proposed that multivariate decoding is enhanced for heterogeneous neural populations with high noise correlations. Extending this proposal to the problem of multivariate decoding with fMRI data, we show that noise correlations between heterogeneous populations of voxels influence MVPA. The same result was obtained across numerous analyses, with the magnitude of noise correlations positively related to classification accuracy. Indeed, MVPA tends to assign greater weights to voxels with high noise correlations. Furthermore, by constructing a simple model that produces artificial BOLD data, we were able to simulate the complementary effects of noise correlations and selectivity on decoding and show that our results generalize across parameter settings.

Why do noise correlations influence multivariate decoding? Most common forms of MVPA work by finding some kind of discrimination boundary or hyperplane in a high-dimensional activity space (Fig 1). Due to variability in BOLD activity, each class to be discriminated is represented by a multivariate distribution in this space, and classification errors result from overlap in these distributions. The intuition is that sensitivity to the noise correlations between voxels coding for different classes allows MVPA to ignore components of variance shared between classes (and thus unhelpful for discriminating between them) by down-weighting dimensions on which this variance loads. This reduces the effective dimensionality of the classification problem, lessening over-fitting given the same amount of training data, and minimizing the overlap between multivariate distributions, thereby improving discrimination between classes.

A recent study [46] similarly considered the influence of noise in BOLD activity on multivariate decoding methods. Specifically, it examined the strategies that are typically used to draw inferences from brain imaging data, and sought to distinguish between “forward models” (e.g., GLMs) evaluating the manner in which experimental variables are encoded in the brain, and “backward models” (e.g., MVPA) seeking to read out experimental variables from brain data. The authors showed that the presence of noise makes the weights from backward models uninterpretable, because these weights are necessarily functions of both signal and noise in the data. In other words, the weight assigned to a given “channel” (or voxel in the case of fMRI) need not only reflect how well it represents the signal of interest—it may be assigned a high weight if the structure of noise in this channel also contributes to the classifier’s effectiveness.

Our study complements and builds upon this and other prior work. Inspired by a computational theory in the literature [36], and in line with [46], we argue that MVPA is effective precisely because it is sensitive to *both* the signal and noise (i.e., selectivity and noise correlations) in patterns of activity across populations of voxels, and the weights assigned to voxels are functions of both of these variables. Our findings go beyond these prior theoretical proposals by providing empirical evidence from a real fMRI dataset and by simulating fMRI data with a range of characteristics. We demonstrate that if a voxel has high noise correlations with voxels selective for the other class, then considering this voxel’s activity allows the classifier to find a better decision boundary (by providing an excellent marker for the noise), thus resulting in the classifier assigning this voxel a higher weight. Furthermore, our analysis scheme highlights a previously overlooked empirical result: The representation of information in human ventral temporal cortex seems to be dominated by a small subset of voxels that are both highly selective for one of the task-relevant categories, and also exhibit high noise correlations with voxels selective for the other category. Thus, at least with the dataset considered here, sensitivity to both selectivity and noise correlations makes MVPA particularly effective at extracting the relevant information. Finally, using a model to simulate different levels of signal and noise in the data, we show that the benefit of noise correlations for decoding is a broadly applicable property of fMRI data, and illustrate how various network parameters influence this finding. Taken together, although other studies have proposed similar ideas from a theoretical perspective, to our knowledge no prior study has validated them at this level of detail, using both empirical and simulated data, and shown how they play out in practice in the context of a widely used multivariate decoding strategy.

In this study, we focused on the classification of face and scene information from ventral temporal cortex as a canonical example of the kind of problem for which MVPA has proven effective. Moreover, this dataset was well suited to an initial exploration of the influence of noise correlations because it contained multiple rest and task runs for each participant allowing for independent definitions of selectivity and noise correlations. We expect that our conclusions will apply to multivariate decoding with brain imaging data more generally. Indeed, the findings from our model—where we observed a similar pattern of results with artificial datasets generated from simulated populations of voxels with a range of physiologically realistic selectivity profiles and noise correlation structures—provide an initial validation of the general applicability of our conclusions. Nevertheless, it will be important for future studies to apply the approach outlined here to other datasets and brain regions.

Another caveat relates to our inability to draw conclusions at the level of neurons from fMRI data. Each voxel in fMRI likely reflects the activity in thousands of neurons, with the exact sampling of neural responses by voxels inaccessible to analysis. Furthermore, the BOLD signal obtained from each voxel reflects a change in blood oxygenation across a broader swath of brain tissue than the neural activity that precipitated this influx of metabolic resources, blurring the link between BOLD contrast and local neural activity. As such, we cannot directly link noise correlations in voxels to noise correlations in neurons, nor draw definitive inferences at the neuronal level from our fMRI results. Nevertheless, at a different level of analysis, our findings support the computational theory that noise correlations can be helpful for extracting information from the brain. As such, although often overlooked, noise correlations should be considered when interpreting the basis and meaning of MVPA.

## Materials and methods

### Participants

Nineteen naïve adults with normal or corrected-to-normal vision participated for monetary compensation. Two participants were excluded because of excessive head motion. The Princeton University Institutional Review Board approved the study protocol and all participants provided informed consent.

### Functional runs

Each participant completed two face/scene “localizer” runs, each of which consisted of an alternating on-off block design, with 18-s blocks of stimulation interleaved with 18-s blocks of “blank” passive fixation. Stimulation blocks contained 12 1-s presentations of either face or scene images (the order of face and scene blocks was counter-balanced across participants), each separated by a 500-ms inter-stimulus interval. Face images consisted of 24 photographs from the NimStim dataset (http://www.macbrain.org/resources.htm, neutral expressions) and scene images consisted of 24 photographs of single houses collected from the internet and stock photograph discs [59]. Images were presented in grayscale, cropped using a circular mask, and subtended 6° of visual angle in radius. In one run, face and scene stimuli were presented in the left visual field, and in the other run, face and scene stimuli were presented in the right visual field. Each run began with a 9-s fixation period and included a total of 12 blocks of stimulation (6 face, 6 scene), which lasted 7m 21s. During blank periods, participants were presented only with a central, white point to fixate (radius = 0.2°). Data from two “rest” runs were also collected for each participant, during a second session. Each rest run had the same duration as the localizer runs, but with only the central fixation point. Participants were instructed to passively view the fixation point without performing any overt task.

### Image acquisition

fMRI data were acquired with a 3T scanner (Siemens Skyra) using a 16-channel head coil. Functional images for both the localizer and rest runs were acquired with a T2* gradient-echo echo-planar imaging sequence (repetition time [TR] = 1.5 s; echo time [TE] = 28 ms; flip angle [FA] = 64°; matrix = 64 x 64; resolution = 3 x 3 x 3.5 mm), with 27 interleaved axial slices aligned to the anterior/posterior-commissure line. TRs during the localizer were time-locked with the presentation of photos. In addition, a high-resolution T1 MPRAGE anatomical scan was acquired for spatial registration. To improve registration, an additional T1 FLASH anatomical scan was acquired at the end of each session, co-planar to the functional scans. To correct for B0-field inhomogeneity, phase and magnitude field maps were collected at the end of all sessions, co-planar to the functional scans and with the same resolution.

### Image analysis

fMRI data were analyzed using FSL (http://fsl.fmrib.ox.ac.uk/fsl/) and Matlab (MathWorks). All functional images were skull-stripped to improve registration, and registered to the anatomical images, and the MNI standard brain. The volumes from the initial 9-s fixation period were removed and the remaining volumes were corrected for slice-acquisition time and head motion, high-pass filtered (100-s period cutoff) and spatially smoothed (5 mm FWHM). Despite potentially blurring the spatial activity patterns used for classification, we applied spatial smoothing for two reasons: First, we wanted our preprocessing and analysis steps to replicate as closely as possible a standard fMRI study in order to quantify how noise correlations influence decoding performance in a situation frequently encountered in cognitive neuroscience. Second, there is debate about the benefits/costs of spatial smoothing for MVPA (benefit being reduced noise, cost being dampened patterns), but the evidence suggests that smoothing with the amount we used is likely beneficial to performance, especially for categorical distinctions [60]. Nevertheless, it will be important in the future to further investigate the impact of smoothing on noise correlations (and how this impacts their utility for feature selection).

### Labeling voxels based on selectivity

Data from the localizer and rest runs were masked to include the temporal occipital fusiform cortex and the parahippocampal gyrus (posterior division), defined anatomically from the Harvard-Oxford cortical atlas in standard MNI space. These regions were chosen because of their general preference for face and scene stimuli, respectively. This mask produced a median of 5875 voxels, which varied less than 2% across participants because of small changes in head position. To identify voxels as face- or scene-selective, we fit a GLM to the BOLD activity observed across the masked ventral temporal voxels during one of the localizer runs (counter-balanced across participants). The GLM contained two main regressors, one for face blocks and the other for scene blocks, as well as six nuisance covariates (one for each motion direction). For each main regressor, a boxcar function lasting the duration of each block was placed at the block onset time, and it was then convolved with a double-gamma hemodynamic response function. The resulting voxelwise parameter estimates for these regressors reflect the average evoked response in each condition. Auto-correlation in the timeseries was corrected with FILM pre-whitening. We labeled voxels as face-selective if the z-scored parameter estimate for the face regressor was greater than the z-scored parameter estimate for the scene regressor, and scene-selective if the opposite was true.

### Calculating noise correlations

We then used the timeseries of BOLD activity for these voxels from the two rest runs to compute their average heterogeneous noise correlations. For each voxel, we calculated the Pearson correlation over time of that voxel with all voxels with the opposite label (e.g., for a face-selective voxel, its correlations with all scene-selective voxels were averaged). Correlations were computed separately for each rest run and averaged across the two runs. Since there were no stimuli or tasks during the rest runs, resulting connectivity can be interpreted as stimulus- or task-independent covariation of variability, i.e. noise correlations.

### Background connectivity

In addition to computing noise correlations from rest runs, we also computed noise correlations from the localizer run used for crossvalidation (counter-balanced across participants). We followed a background connectivity approach [2, 44]. After preprocessing, the BOLD activity in the localizer run was scrubbed of nuisance and stimulus-evoked variance using two GLMs. The first (nuisance) model contained regressors for the global mean activity, six motion correction parameters obtained from preprocessing, and the activity from four seeds in white matter and from four seeds in the ventricles. Residuals from the nuisance model served as input to the second (evoked) model. As described earlier, each localizer run consisted of 6 identically structured blocks per category. To precisely capture the averaged evoked response for each category, we created 48 finite impulse response (FIR) regressors—one for each volume of a full 72-s cycle of two blocks (face-blank-scene-blank). Each regressor had a constant height of 1 at one specific volume of every block, and height of 0 elsewhere. That is, one regressor modeled the average evoked response in the first volume of all face blocks, another the second volume, and so forth. We used an FIR model because it avoids *a priori* assumptions about the shape and timing of the hemodynamic response. Correlations computed over the residuals from the evoked model, just as described above for the rest runs, allowed us to assess heterogeneous noise correlations orthogonal to global noise sources and stimulus-evoked responses.

### MVPA

For classification analyses, we used the Princeton Multi-Voxel Pattern Analysis Toolbox (www.pni.princeton.edu/mvpa). Specifically, we used subject-specific logistic regression classifiers penalized using L2-norm regularization (penalty = 1; preliminary analyses showed negligible influence of this parameter on the qualitative pattern of our results). We performed three-way (face/scene/blank) classification by learning weights for three logistic regression models during the training phase (discriminating TRs as face vs. not, scene vs. not, and blank vs. not, respectively) and then generating guesses during the test phase by labeling each TR according to the model with maximal output evidence. We verified in preliminary analyses that including the blank blocks and performing multi-way classification (as opposed to binary face vs. scene classification) did not affect the pattern of results.

To quantify classification accuracy, we averaged the results of 6-fold cross-validation. The classifier in each fold was trained on 5/6^th^ of the data and tested on the left-out 1/6^th^ of the data. Because only one localizer run was used for this cross-validation (the other was used to independently define selectivity), these divisions of the data into training and test sets occurred in the same fMRI run. Data from the same run can have dependencies, both locally when activity in the previous block spills over into the current block, and globally as a result of non-task factors like head motion or arousal. Despite this, our within-run approach was unbiased. With respect to local dependencies, all conditions being classified were present in each run and alternated between each other, and thus any spill-over (into a period with a different label) would hurt performance. With respect to global dependencies, because again the full design existed within each run (and training/test sets), any general factors would apply to all conditions and not systematically support classification between conditions. Chance classification accuracy was calculated empirically by randomly permuting the category labels across TRs in the localizer run before performing MVPA (block-level scrambling produced identical results). This process was repeated 10,000 times for each participant, and the average classifier accuracy across permutations and participants provided the baseline level of performance that would be expected due to chance.

### Model simulations

We developed a simple model of face/scene selectivity in BOLD data from human ventral temporal cortex to examine the separate influence of noise correlations and selectivity on MVPA. We simulated a set of 30 face-selective voxels and 30 scene-selective voxels. Mean activity in each of the face-selective voxels took on a larger value in response to a face stimulus (M_FF_) than in response to a scene stimulus (M_FS_), and vice versa for scene-selective voxels (M_SF_ and M_SS_). Based on the empirical dataset, these parameters were set at baseline to (in arbitrary units): M_FF_ = 708, M_FS_ = 705, M_SS_ = 740 and M_SF_ = 735. Pairwise correlations in the activity of voxels took on different values within the face-selective pool of voxels (c_FF_), within the scene-selective pool of voxels (c_SS_), and across the two pools (c_FS_, c_SF_). These parameters were set at baseline to: c_FF_ = 0.2, c_SS_ = 0.2, and c_FS_ = c_SF_ = 0. The activity of voxels in both pools had the same effective variance (*σ*^2^), set at baseline to *σ* = 12. For each of 100 simulated participants, we independently sampled voxel data from these distributions for 72 face and 72 scene timepoints. The resulting voxel timecourses were convolved with a canonical hemodynamic response function.

Model parameters were modulated to examine the influence of selectivity and noise correlations on classification accuracy. These parameters are listed below for each of the simulations, grouped by the subpanel of the figure containing the results: (1) Fig 9A: c_FS_ and c_SF_ were linearly varied between 0 and 0.22; “high selectivity”, M_FF_, M_FS_, M_SS_ and M_SF_ were set to the baseline values; “med selectivity”, M_FF_ = 708, M_FS_ = 706, M_SS_ = 740 and M_SF_ = 736; “low selectivity”, M_FF_ = 707, M_FS_ = 706, M_SS_ = 739 and M_SF_ = 736. All other parameters were set to baseline values. (2) Fig 9B: c_FS_ and c_SF_ were linearly varied between 0 and 0.22; “low variance”, *σ* = 9; “med variance”, *σ* was set to the baseline value; “high variance”, *σ* = 15. All other parameters were set to baseline values. (3) Fig 9C: For each model participant and voxel, we randomly drew from a Gaussian distribution with vanishing mean and standard deviation of 100, and added this value to the baseline mean response of the voxel; we also randomly varied the population covariance matrix according to a Gaussian distribution with vanishing mean and standard deviation equal to 10% of the baseline value of the corresponding matrix element.

## Supporting information

S1 Fig MVPA without regularization. Classification accuracy decreased across the board when regularization was turned off, but remained better for voxels with high (green) vs. low (blue) noise correlations, with a similar interaction by bin size. Columns represent means and error bars represent SEM across participants. The dashed gray line denotes permuted chance. ***p < 0.001, **p < 0.01.
(TIF)
