# Supplementary figures and images for: Noise correlations in the human brain and their impact on pattern classification
Source: PLoS Comput Biol. 2017 Aug 25;13(8):e1005674. doi: 10.1371/journal.pcbi.1005674 (PMC5589258; doi:10.1371/journal.pcbi.1005674)

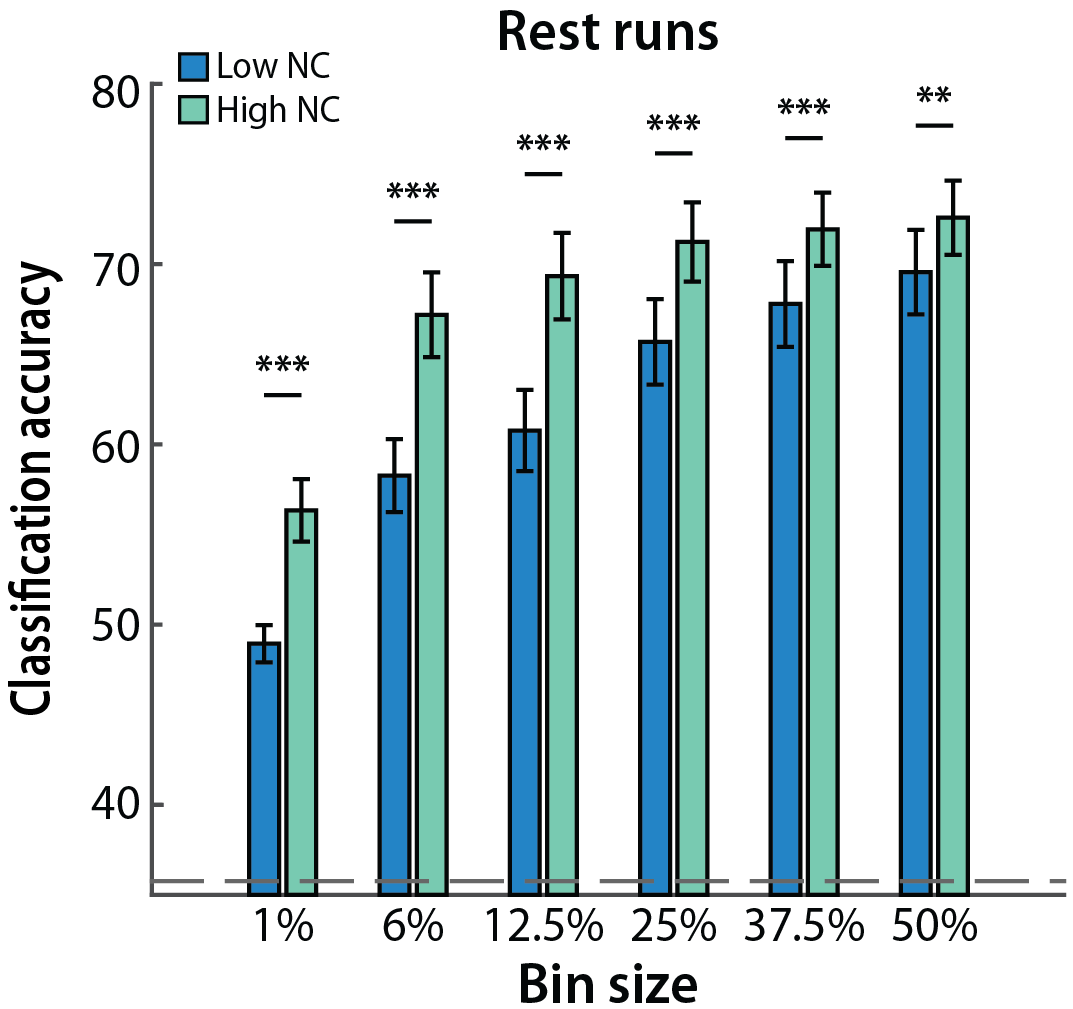

Supplement: S1 Fig — Classification accuracy decreased across the board when regularization was turned off, but remained better for voxels with high (green) vs. low (blue) noise correlations, with a similar interaction by bin size. Columns represent means and error bars represent SEM across participants. The dashed gray line denotes permuted chance. ***p < 0.001, **p < 0.01. (TIF) [file pcbi.1005674.s001.tif]
